# The association between deficiency of nutrient intake and resting metabolic rate in overweight and obese women: a cross-sectional study

**DOI:** 10.1186/s13104-021-05582-z

**Published:** 2021-05-12

**Authors:** Seyedeh Forough Sajjadi, Atieh Mirzababaei, Afsoun Abdollahi, Farideh Shiraseb, Khadijeh Mirzaei

**Affiliations:** 1 Department of Community Nutrition, School of Nutritional Sciences and Dietetics, Tehran University of Medical Sciences (TUMS), P.O. Box: 14155-6117, Tehran, Iran; 2 Department of Nutrition Science, Purdue University, West Lafayette, IN 47907 USA

**Keywords:** Resting metabolic rate, Fat-free mass, Obesity, Overweight, The double burden of malnutrition, Nutrient adequacy ratio

## Abstract

**Objective:**

The double burden of malnutrition is an emerging public health concern nowadays which a correlation with obesity. This study aimed to examine the relationship between resting metabolic rate (RMR) and dietary intake of zinc, vitamin C, and riboflavin in overweight and obese women.

**Results:**

The RMR/FFM showed a significant association with riboflavin (β = 1.59; 95% CI 1.04–23.26, P = 0.04) and zinc (β = 0.78; 95% CI 1.04–4.61, P = 0.03) in the crude model. Moreover, differences in vitamin C and RMR/FFM was marginal significant (β = 0.75; 95% CI 0.95–4.77, P = 0.06). After adjusting for confounders the riboflavin association change to marginal significance (β = 1.52; 95% CI 0.91–23.04, P = 0.06). After controlling for potential confounders, the associations change between zinc and RMR/FFM (β = 0.66; 95% CI 0.78–4.86, P = 0.15) and between RMR/FFM and vitamin C (β = 0.48; 95% CI 0.66–3.96, P = 0.28). Our study showed a significant association between dietary intake of zinc, riboflavin, and vitamin C and change in RMR/FFM in overweight and obese women.

## Introduction

Obesity rates are growing globally [1]. More than 26% of the Iranian adult community is currently obese [2]. Females were much more affected than males [3, 4]. Obesity and its associated metabolic disorders develop when energy intake is more than energy expenditure; this can be caused by diminished physical activity, the disability of the central nervous system to down-regulate the ingestion of high-calorie foods, or appetite [5]. Although various factors contribute to the etiology of obesity, sedentary lifestyles, and unhealthy eating habits are among the principal contributors to the world obesity epidemic [6]. 60–75% of total energy expenditure is correlated with resting metabolic rate (RMR), which is an important part of daily energy consumption [7]. Weight gaining and obesity may be associated with low RMR [8].

Diet quality can be evaluated to better understand overall eating patterns [9]. Poor diet quality is a significant factor in the development of many chronic diseases, including obesity [10]. Investigations revealed, however, intake of vegetables and fruit is associated with a lower risk of obesity [11], inappropriate food behavior contributes to obesity and contributes to vitamin deficiency. Studies revealed that most vitamins are inadequate in obese individuals [12]. The double burden of malnutrition (DBM) is an emerging public health concern nowadays that happens as an inevitable consequence of nutritional transition [13]. The coexistence of overnutrition and undernutrition is often referred to as the DBM [14]. Nutrient adequacy ratio (NAR) and mean adequacy ratio (MAR), known as healthier diet quality indices. Higher scores in diet quality inversely connected with body mass index (BMI), and obesity [15]. Similarly, zinc and vitamin C are closely related to adiposity [16, 17]. In particular, some diet with a high score of diet quality like the Mediterranean dietary pattern has been reported to be inversely connected with BMI, and waist circumference [18].

To the best of our knowledge, this is the first study to investigate the relationship between deficiency of nutrient intakes such as zinc, vitamin C, and riboflavin and RMR in the adult women population. Accordingly, this study was performed to examine the NAR with RMR/FFM among a group of Iranian adult women.

## Main text

### Materials and methods

#### Study population

This cross-sectional research was performed on 293 adult women aged between 18 and 48 years who were selected by a multistage cluster random sampling method that had been referred to health centers in Tehran recruited. Participants were enrolled in the study according to inclusion and exclusion criteria. Inclusion criteria were: general health, overweight and obese women with BMI in the range of 25–40 kg/m^2^. The exclusion criteria were as follows: regular use of medicine, history of hypertension, cardiovascular diseases, and other chronic diseases, alcohol consumption, smoking, pregnancy, lactation period, and menopause. Furthermore, those who had been following an arbitrary special dietary regimen, and also those with any significant body weight fluctuations over the past 1 year and energy intakes lower than 800 kcal/day or higher than 4200 kcal/day were excluded.

#### Energy expenditure measurements

RMR was measured by indirect calorimetry (spirometer METALYZERR 3B-R3, Cortex Biophysik GmbH, Leipzig, Germany). According to the manufacturer’s instructions, gas ventilation and exchange is calibrated before each test. RMR is evaluated by measuring the amount of O_2_ consumed and CO_2_ produced. The RMR was assessed in the morning, after a comfortable night’s sleep, and following a 10–12 h fast. Participants were asked to avoid caffeine or alcohol consumption and severe exercise for a day before RMR measurements. After reclining in a steady-state and a supine position in a quiet room, the RMR was measured for 30 min. The respiratory exchange ratio and oxygen uptake (VO_2_) were analyzed within the middle 20 min of the resting period. Predictive RMR was determined using the Harris–Benedict equation, which considers the weight, height, and age of participants.

### Body composition measurement

Body composition, including weight, BMI, fat mass, and fat-free mass (FFM) were acquired using a multi-frequency bioelectrical impedance analyzer InBody 770 scanner (Inbody Co., Seoul, Korea According to the manufacturer’s instructions, participants removed their shoes, coats, and sweaters, and stood on the balance scale in bare feet, and grasped the handles of the machine.

### Biochemical assessment and hormonal assay

Metabolic health was assessed using the metabolic parameters that measured following standard chemical procedures. A 12-h fasting venous blood sample was used to measure all biochemical markers. Serum glucose was evaluated by a colorimetric method based on the GOD-PAP method. Serum insulin concentrations were analyzed by enzyme-linked immunosorbent assay (ELISA) method (Human insulin ELISA kit, Monobind Inc., Lake Forest, USA).

### HOMA and QUICKI calculations

Insulin resistance was estimated by homeostasis model assessment (HOMA). The HOMA was calculated according to the following equation: HOMA = [Fasting Plasma Glucose (mmol/L) × Fasting Plasma Insulin (mIU/L)]/22.5 [19]. Insulin sensitivity quantitative insulin sensitivity check index (ISQUICKI) was assessed by: ISQUICKI = 1/[log (fasting insulin) + log (fasting glucose) [20].

### Dietary intake assessment

Dietary intake data of the past year were obtained using a validated semi-quantitative food-frequency questionnaire (FFQ) [21], comprised of 168-item a trained nutritionist administered these FFQ. The FFQ consisted of a list of foods with standard serving sizes. Participants were asked to report their frequency and amount of each food item consumed during the previous year. Portion sizes of the consumed foods were converted to grams using household measurements [22]. Nutritionist IV computer software was used for the nutrient analysis of the diets. The database of this software was modified for Iranian foods.

### Nutrient adequacy ratios (NAR)

For calculating the NAR, the ratio of daily individual intakes to the standard recommended amounts for the subject’s sex and age category was used. The standard recommended amounts are based on RDA (Recommended daily allowances) [23]. We calculated the NAR for three key nutrients, including zinc, vitamin C, and riboflavin according to the above-mentioned method. The prevalence of nutrient deficiency was estimated using NAR. NAR lower than one is considered as a deficiency.

### Assessment of other covariates

International physical activity questionnaire (IPAQ, short form) were obtained by using an interview-based questionnaire from all participants about all the vigorous and moderate elements over the last 7 days, considering the time spent on these activities for height measurements, subjects were in a standing position without shoes, in contact with the wall with their head, shoulders, heels, and hips, and their height was recorded to the nearest 0.1 cm.

### Statistical analysis

All statistical analysis was performed using the IBM SPSS software version 22.0 (SPSS, Chicago, IL, USA), and P-values less than 0.05 were considered statistically significant. The normal distribution of data was checked by the Kolmogorov–Smirnov test. An independent sample t test was used for assessed differences between groups with the low and standard intake of nutrients. RMR/FFM was analyzed after adjusting for FFM. The differences between RMR/FFM groups and dietary intake of nutrients were assessed by the binary logistic regression were performed to adjust for confounders effects such as age, energy intake, and physical activity (METs/day). Results were presented as odds ratios (ORs) and 95% confidence intervals (CIs) compared with the RMR groups.

### Results

#### Study population characteristics

A total of 293 healthy overweight and obese women were enrolled. The mean age, height, weight, and BMI of the study participants were 36.39 years (SD = 8.71), 161.84 cm (SD = 5.85), 80.22 kg (SD = 11.28), and 30.77 kg/m^2^ (SD = 3.79), respectively (Table 1) the mean body composition, RMR components, biochemical and anthropometric characteristics of subjects are shown in Table 1.

**Table 1:** Study of population characteristics

Parameters	Minimum	Maximum	Mean ± SD
Age (years)	18	56	36.39 ± 8.71
Height (cm)	147.50	179.00	161.84 ± 5.85
Weight (kg)	59.50	122.40	80.22 ± 11.28
BMI (kg/m^2^)	25.00	40.70	30.77 ± 3.79
RMR parameters			
RMR measure (kcal/day)	952.00	2467.00	1569.60 ± 255.70
RQ	0.73	0.99	0.85 ± 0.04
RMR/FFM (kcal/day/kg)	21.50	45.86	33.74 ± 4.48
V. O_2_	0.14	0.35	0.22 ± 0.036
V. CO_2_	0.01	0.30	0.19 ± 0.034
Body composition parameters			
Body fat mass (kg)	19.40	59.40	33.52 ± 7.74
Fat free mass (kg)	35.30	67.70	46.68 ± 5.50
Blood parameters			
FBS (mg/dl)	67.00	137.00	87.39 ± 9.76
Insulin (mIU/ml)	6.67	65.89	15.59 ± 6.02
HOMA	1.29	16.59	3.40 ± 1.52
ISQUICKI	0.39	0.68	0.54 ± 0.04

Data are indicated as mean ± SD otherwise indicated

*RMR* resting metabolic rate; *RQ* respiratory quotient; *FFM* fat-free mass; *V. O2* oxygen consumption; *V. CO2* carbon dioxide consumption; *FBS* fasting blood sugar; *HOMA* homeostasis model assessment; *ISQUICKI* insulin sensitivity quantitative insulin sensitivity check index

n = 293

#### Participant’s characteristics between standard and deficiency of daily nutrient intakes

Dietary intake of three nutrients including riboflavin, vitamin C, and zinc were categorized based on nutrient adequacy ratios (NAR) and divided into two groups, standard and deficiency (Table 2). The RMR indicated a significant association with zinc (P = 0.001), which demonstrates people who consume higher zinc had higher RMR. Moreover, other factors like RMR/FFM (P = 0.06), V. O_2_ (P = 0.001), V. CO_2_ (P = 0.007), body fat mass (P = 0.02), FFM (P = 0.006), height (P = 0.005), and weight (P = 0.006) had a significant relationship with zinc. Besides, riboflavin had a significant association with body fat mass (P = 0.05), however, there were showed no relationship between vitamin C and participant characteristics (P > 0.05).

**Table 2:** Participants’ characteristics between standard and deficiency of daily nutrient intakes

Parameters	Nutrient								
	Vitamin C			Zinc			Riboflavin		
	NAR < 1	NAR ≥ 1	P-value	NAR < 1	NAR ≥ 1	P-value	NAR < 1	NAR ≥ 1	P-value
	n = 30	n = 250		n = 36	n = 244		n = 11	n = 269	
	10.2%	85.3%		12.3%	83.3%		3.8%	91.8%	
Age (years)	34.90 ± 8.62	63.60 ± 8.53	0.30	38.05 ± 9.13	36.18 ± 8.44	0.22	31.63 ± 8.41	36.62 ± 8.50	0.08
Height (cm)	161.63 ± 6.27	161.32 ± 5.82	0.78	158.78 ± 6.33	161.74 ± 5.70	**0.005**	160.27 ± 8.16	161.40 ± 5.76	0.53
Weight (kg)	77.54 ± 9.28	80.32 ± 11.13	0.19	75.35 ± 8.78	80.71 ± 11.10	**0.006**	75.61 ± 14.41	80.20 ± 10.80	0.17
BMI (kg/m^2^)	29.78 ± 3.29	30.87 ± 3.78	0.13	29.91 ± 3.40	30.88 ± 3.78	0.15	29.30 ± 4.03	30.81 ± 3.72	0.19
RMR parameters									
RMR measure (kcal/day)	1500.2 ± 307.28	1580.2 ± 247.49	0.17	1437.5 ± 288.33	1591.7 ± 244.25	**0.001**	1450.6 ± 301.44	1576.5 ± 252.59	0.10
RQ	0.86 ± 0.03	0.85 ± 0.04	0.16	0.86 ± 0.042	0.85 ± 0.041	0.39	0.858 ± 0.03	0.854 ± 0.04	0.34
RMR/FFM (kcal/day/kg)	00.04 ± 5.60	33.92 ± 4.36	0.32	32.50 ± 5.10	34.02 ± 4.39	0.06	31.79 ± 2.93	33.91 ± 4.54	0.12
V. O_2_	0.21 ± 0.04	0.22 ± 0.03	0.13	0.20 ± 0.04	0.22 ± 0.03	**0.001**	0.20 ± 0.04	0.22 ± 0.03	0.13
V. CO_2_	0.18 ± 0.04	0.19 ± 0.03	0.22	0.17 ± 0.04	0.19 ± 0.03	**0.007**	0.17 ± 0.04	0.19 ± 0.03	0.16
Body composition parameters									
Body fat mass (kg)	31.80 ± 6.66	33.65 ± 7.77	0.21	30.79 ± 6.66	33.84 ± 7.74	**0.02**	29.15 ± 7.10	33.63 ± 7.66	**0.05**
Fat free mass (kg)	45.63 ± 5.26	46.76 ± 5.44	0.28	44.27 ± 4.74	46.98 ± 5.44	**0.006**	45.55 ± 7.70	46.69 ± 5.33	0.49
Blood parameters									
FBS (mg/dl)	86.50 ± 10.59	87.51 ± 9.64	0.60	87.93 ± 11.62	87.30 ± 9.34	0.73	83.30 ± 7.33	87.57 ± 9.81	0.17
Insulin (mIU/ml)	14.75 ± 4.77	15.70 ± 6.30	0.44	15.37 ± 4.15	15.62 ± 6.41	0.83	18.28 ± 10.03	15.47 ± 5.93	0.40
HOMA	3.17 ± 1.17	3.42 ± 1.59	0.41	3.35 ± 1.05	3.40 ± 1.61	0.84	3.81 ± 2.33	3.38 ± 1.50	0.38
ISQUICKI	0.55 ± 0.045	0.54 ± 0.049	0.49	0.540 ± 0.03	0.545 ± 0.05	0.58	0.53 ± 0.05	0.54 ± 0.04	0.70

Bold value reflect significant value, P-value < 0.05

Data are indicated as mean ± SD otherwise indicated

*RMR* resting metabolic rate; *RQ* respiratory quotient; *FFM* fat-free mass; *V. O2* oxygen consumption; *V. CO2* carbon dioxide consumption; *FBS* fasting blood sugar; *HOMA* homeostasis model assessment; *ISQUICKI* insulin sensitivity quantitative insulin sensitivity check index

n = 293

P-values are from independent sample t test

#### Association of riboflavin, vitamin C, and zinc with RMR/FFM among obese women

Table 3 shows multivariate-adjusted models for the prevalence of higher RMR/FFM across the median dietary intake of riboflavin, vitamin C, and zinc. The RMR/FFM showed a significant association with riboflavin, vitamin C, and zinc in the crude model. For riboflavin, in the crude model before adjustment for the confounders, showed a statistically significant relationship (β = 1.59; 95% CI 1.04–23.26, P = 0.04). After controlling for the potential confounders, the association change to marginal significance (β = 1.52; 95% CI 0.91–23.04, P = 0.06). Moreover, differences in vitamin C and RMR/FFM was marginal significant (β = 0.75; 95% CI 0.95–4.77, P = 0.06). But after controlling for the potential confounders, the association disappeared (P = 0.28). Differences in zinc and RMR/FFM were also significant in the crude model (β = 0.78; 95% CI 1.04–4.61, P = 0.03), but after adjustment for the potential confounders, the association disappeared (P = 0.15).

**Table 3:** Association of riboflavin, vitamin C and zinc and RMR/FFM among obese women

RMR/FFM	β	OR (95% CI)	P-value
Riboflavin			
Crude model	1.59	4.93 (1.04–23.26)	**0.04**
Model 1	1.75	5.80 (1.21–27.77)	**0.02**
Model 2	1.71	5.55 (1.11–27.59)	**0.03**
Model 3	1.52	4.58 (0.91–23.04)	0.06
Vitamin C			
Crude model	0.75	2.13 (0.95–4.77)	0.06
Model 1	0.82	2.27 (1.01–5.13)	**0.04**
Model 2	0.79	2.20 (0.93–5.19)	0.07
Model 3	0.48	1.62 (0.66–3.96)	0.28
Zinc			
Crude model	0.78	2.19 (1.04–4.61)	**0.03**
Model 1	0.71	2.04 (0.96–4.33)	0.06
Model 2	0.71	2.04 (0.87–4.76)	0.09
Model 3	0.66	1.95 (0.78–4.86)	0.15

Bold value reflect significant value, P-value < 0.05

Model 1: adjusted for age. Model 2: further adjusted for energy intake. Model 3: further adjusted for physical activity (METs/day)

P-values are from binary logistic regression

## Discussion

The results of this study showed a significant association between riboflavin intake and the RMR/FFM. RMR was distinct in standard or deficiency consumption of riboflavin. There was not any significant association between vitamin C, zinc and RMR/FFM.

The findings of the current study indicate that there is no association between the amount of zinc consumed and RMR. However, other studies demonstrated that zinc has different functions in the metabolism of energy and works as a component of several enzymes crucial to the metabolism of carbohydrates, proteins, and lipids and metabolism of hormones that take part in the progress of obesity, especially insulin, and seems to be connected with the mechanisms of insulin resistance usually present among obese people [11, 24–28]. Previous investigations recommend a negative association between RMR and insulin resistance [29, 30]. Subjects with obesity and impaired glucose tolerance showed higher RMR levels than those with obesity and normal glucose tolerance [31]. This discrepancy in the findings may be due to the limitations of the present study such as participants in the same-sex sample and assessing dietary intakes from FFQ. This study found no significant association between vitamin C and RMR. Some studies have shown that vitamin C administration significantly decreased RMR [33]. A probable mechanism could be due to the role of ascorbic acid in the expression of genes involved in adipogenesis, metabolism of glucocorticoids [12, 34] and inflammatory response. The result of this study is in line with previous studies which showed that the increase in blood vitamin C concentrations associated with the change in RMR/FFM [32]. This finding was consistent with a previous observation that by Selman Colin that showed the vitamin C supplementation did not affect daily energy expenditure or resting metabolism. This finding strongly recommends that antioxidant effects of the vitamin were not being compensated for by modulations in the rate of oxidative metabolism, which might affect total rates of reactive oxygen species product [35].

In this study, we also found that women who consumed higher riboflavin were more likely to have higher RMR. Considering the dietary restrictions on food intake or common dietary mistakes perceived among obese people, changes in the micronutrient intake leading to their deficiency are possible. Suitable riboflavin content is necessary to perform the effector function of macrophages with inhibition proliferation, intensification of apoptosis incidence, and also the reduction in phagocytosis efficiency [9]. Furthermore, resting reactive oxygen species production was raised while respiratory burs, a key ingredient of intracellular killing, were destroyed. Considering the significant function of adipocytes in the creation of obesity-related chronic inflammation to be justifiable to verify the influence of riboflavin deficiency on adipocytes function in the context of pro-inflammatory activation [36, 37]. That seems for future need more attention to the intake of sufficient micronutrients according to guidelines for preventing decreasing RMR so that reduce overweight and obesity. According to this study, more attention needs for riboflavin rich foods.

### Conclusions

In conclusion, we could find a significant association between dietary intake of riboflavin, and change in RMR/FFM in overweight and obese women, however after controlling for ranges of potential confounding factors the statically meaningful for zinc and vitamin C disappear.

### Limitation

The major limitation of this study was the participants in the same-sex sample that it is not possible to generalize the results to men population. Because of the study type, cross-sectional study, we could not determine the causality. Another limitation for assessing dietary intakes from FFQ is misclassification. Albeit we controlled for the effect of the potential confounder by the statistical methods, because of unknown confounder cannot be excluded residual confounding will affect.

## Data Availability

Participants in this study did not agree to the public sharing of their data so supporting data is not available.

## Abbreviations

RMR: Resting metabolic rate
DBM: Double burden of malnutrition
NAR: Nutrient adequacy ratio
MAR: Mean adequacy ratio
BMI: Body mass index
FFM: Fat-free mass
ELISA: Enzyme-linked immuno-sorbent assay
HOMA: Homeostasis model assessment
ISQUICKI: Insulin sensitivity quantitative insulin sensitivity check index
FFQ: Food frequency questionnaire
RDA: Recommended daily allowances
IPAQ: International physical activity questionnaire
RQ: Respiratory quotient
FBS: Fasting blood sugar
